# Comparative Genomics of the Genus *Porphyromonas* Identifies Adaptations for Heme Synthesis within the Prevalent Canine Oral Species *Porphyromonas cangingivalis*

**DOI:** 10.1093/gbe/evv220

**Published:** 2015-11-13

**Authors:** Ciaran O’Flynn, Oliver Deusch, Aaron E. Darling, Jonathan A. Eisen, Corrin Wallis, Ian J. Davis, Stephen J. Harris

**Affiliations:** ^1^The WALTHAM Centre for Pet Nutrition, Waltham-on-the-Wolds, United Kingdom; ^2^The ithree Institute, University of Technology Sydney, Ultimo, New South Wales, Australia; ^3^Department of Evolution and Ecology, University of California, Davis; ^4^Department of Medical Microbiology and Immunology, University of California, Davis; ^5^UC Davis Genome Center, University of California, Davis

**Keywords:** periodontal disease, comparative genomics, *Porphyromonas*, canine, plaque

## Abstract

Porphyromonads play an important role in human periodontal disease and recently have been shown to be highly prevalent in canine mouths. *Porphyromonas cangingivalis* is the most prevalent canine oral bacterial species in both plaque from healthy gingiva and plaque from dogs with early periodontitis. The ability of *P. cangingivalis* to flourish in the different environmental conditions characterized by these two states suggests a degree of metabolic flexibility. To characterize the genes responsible for this, the genomes of 32 isolates (including 18 newly sequenced and assembled) from 18 Porphyromonad species from dogs, humans, and other mammals were compared. Phylogenetic trees inferred using core genes largely matched previous findings; however, comparative genomic analysis identified several genes and pathways relating to heme synthesis that were present in *P. cangingivalis* but not in other Porphyromonads. *Porphyromonas cangingivalis* has a complete protoporphyrin IX synthesis pathway potentially allowing it to synthesize its own heme unlike pathogenic Porphyromonads such as *Porphyromonas gingivalis* that acquire heme predominantly from blood. Other pathway differences such as the ability to synthesize siroheme and vitamin B12 point to enhanced metabolic flexibility for *P. cangingivalis*, which may underlie its prevalence in the canine oral cavity.

## Introduction

The genus *Porphyromonas* is considered one of the most important in human periodontitis and a large amount of research has been undertaken into the role of *Porphyromonas gingivalis* in particular (Lamont et al. 1995; Griffen et al. 1998; Holt et al. 1999; Holt and Ebersole 2005)*.* The central role of *P. gingivalis* in the progression of human periodontitis has led to its classification as a “keystone pathogen” (Darveau et al. 2012) which influences the composition of the oral microbiome even when present at low levels. Recent surveys of the canine oral microbiota (Dewhirst et al. 2012; Davis et al. 2013) have hinted at a role for Porphyromonads in canine oral health and periodontitis. For instance, *Porphyromonas gulae* which is closely related to *P. gingivalis* (∼98% sequence identity) based on 16S rDNA sequencing data (Fournier et al. 2001) is the second most common Porphyromonad measured by 16S rDNA sequencing of plaque from dogs with early periodontitis (Davis et al. 2013). In addition, *Porphyromonas cangingivalis* showed the highest relative abundance of 16S rDNA sequences of any canine oral species, with an average of 10.5% in plaque from healthy gums and 4.6% in plaque from periodontitis samples and reached as high as 25% relative abundance in both health and disease samples (Davis IJ, unpublished data). The ability of *P. cangingivalis* to predominate in both health and disease environments suggests that it is both metabolically flexible enough to colonize in health and also able to compete against other *Porphyromonas* spp. in a disease environment (Davis et al. 2013). A comparison of the genes present in the *P. cangingivalis* genome compared with those found in other Porphyromonads therefore has the potential to identify genes that support that metabolic flexibility. The genomes of various Porphyromonads from dog, human, and other mammals were compared to look at their relatedness and for differences in gene content. The ultimate aim is to better understand the role of the Porphyromonads found in canine plaque and generate information to assist the future development of treatments to reduce periodontitis in dogs.

## Materials and Methods

### Genomes Used

This study used 32 *Porphyromonas* genomes including representatives from all publicly available *Porphyromonas* species (table 1). Of the 32 genomes, 18 were of canine oral isolates previously sequenced and assembled by the authors (Coil et al. 2015). The remaining 14 were complete or partially assembled genomes downloaded from National Center for Biotechnology Information (NCBI) (Sayers et al. 2009). Species characteristics (assembly statistics, host species, host sites, and disease associations) (table 1) for the canine isolates were acquired from previous studies (Sayers et al. 2009; Davis et al. 2013), for noncanine strains from NCBI or individual genome release papers (Hirasawa and Takada 1994; Summanen et al. 2005, 2009; Sakamoto and Ohkuma 2013; Sakamoto et al. 2013). In addition to the *Porphyromonas* genomes, four further *Bacteroidales* genomes were used as outgroups in the phylogenetic analyses. These were *Bacteroides fragilis*, *Paludibacter propionicigenes*, *Tannerella forsythia*, and the canine isolate *Porphyromonadaceae* [G-1] sp. COT-184.

**Table 1:** Summary of *Porphyromonas* Genomes

Isolate	Accession	Number of Contigs	Size (Mb)	GC%	Proteins	Host	Site	Health Status of Mouth at Point of Isolation	Disease Association of Species (Health/Disease)
*Porphyromonas asaccharolytica* DSM 20707 uid66603	NC_015501	1	2.19	52.5	1,699	Human	Nonoral—puss	—	—
*Porphyromonas asaccharolytica* PR426713P I uid61039	AENO00000000	58	2.20	52.3	1,655	Human	Nonoral	—	—
*Porphyromonas bennonis* DSM 23058	AQWR00000000	87	2.02	56.3	1,407	Human	Nonoral—skin	—	—
*Porphyromonas catoniae* F0037 uid183770	AMEQ00000000	44	2.11	51.0	1,855	Human	Oral	—	—
*Porphyromonas endodontalis* ATCC 35406 uid55449	ACNN00000000	37	2.06	47.5	1,965	Human	Oral	—	—
*Porphyromonas gingivalis* ATCC 33277 uid58879	NC 010729	1	2.35	48.4	2,089	Human	Oral	—	D
*Porphyromonas gingivalis* TDC60 uid67407	NC 015571	1	2.34	48.3	2,217	Human	Oral	—	D
*Porphyromonas gingivalis* W50 uid180461	AJZS00000000	104	2.24	48.3	2,016	Human	Oral	—	D
*Porphyromonas gingivalis* W83 uid57641	NC 002950.2	1	2.34	48.3	1,909	Human	Oral	—	D
*Porphyromonas oral taxon* 279 F0450 uid174237	ALKJ00000000	51	2.27	55.5	1,729	Human	Oral	—	—
*Porphyromonas somerae* DSM 23386	AQVC00000000	95	2.36	47.0	1,922	Human	Nonoral—leg ulcer	—	—
*Porphyromonas uenonis* 60 3 uid55869	ACLR00000000	250	2.24	52.5	1,977	Human	Nonoral—vagina	—	—
*Porphyromonas cangingivalis* COT-109 OH1379	JQJF00000000	21	2.36	47.7	1,708	Dog	Oral	Gingivitis	H
*Porphyromonas cangingivalis* COT-109 OH1386	JQJD00000000	65	2.44	47.6	1,771	Dog	Oral	Gingivitis	H
*Porphyromonas canoris* COT-108 OH1224	JQZX00000000	21	2.31	44.6	1,708	Dog	Oral	PD1	—
*Porphyromonas canoris* COT-108 OH1349	JRAH00000000	43	2.33	44.6	2,153	Dog	Oral	Gingivitis	—
*Porphyromonas canoris* COT-108 OH2762	JQZV00000000	14	2.20	44.7	1,612	Dog	Oral	Gingivitis	—
*Porphyromonas canoris* COT-108 OH2963	JRAP00000000	21	2.18	44.8	1,955	Dog	Oral	PD1	—
*Porphyromonas cansulci* JCM 13913	BAOV00000000	89	2.11	45.4	2,180	Dog	Oral	—	D
*Porphyromonas crevioricanis* COT-253 OH1447	JQJC00000000	30	2.16	45.4	1,607	Dog	Oral	PD1	D
*Porphyromonas crevioricanis* COT-253 OH2125	JQJB00000000	14	2.11	45.4	1,583	Dog	Oral	Gingivitis	D
*Porphyromonas gingivicanis* COT-022 OH1391	JQZW00000000	19	1.98	42.7	1,433	Dog	Oral	Gingivitis	—
*Porphyromonas gulae* I COT-052 OH1355	JRAG01000000	40	2.34	48.6	1,847	Dog	Oral	Gingivitis	D
*Porphyromonas gulae* I COT-052 OH3471	JRAQ01000000	44	2.37	48.6	1,877	Dog	Oral	Health	D
*Porphyromonas gulae* I COT-052 OH4946	JQZY00000000	34	2.38	48.5	2,127	Dog	Oral	PD1	D
*Porphyromonas gulae* II COT-052 OH2857	JRFD01000000	53	2.33	48.7	1,874	Dog	Oral	Gingivitis	D
*Porphyromonas gulae* II COT-052 OH3856	JRAT00000000	31	2.39	48.5	1,904	Dog	Oral	Health	D
*Porphyromonas macacae* COT-192 OH2859	JRFA00000000	32	2.36	43.2	1,843	Dog	Oral	Gingivitis	D
*Porphyromonas* sp. COT-239 OH1446	JRAO00000000	37	1.96	53.9	1,412	Dog	Oral	PD1	D
*Porphyromonas* sp. COT-290 OH0860	JRAR01000000	82	2.34	49.7	1,619	Dog	Oral	Gingivitis	H
*Porphyromonas* sp. COT-290 OH3588	JRFC01000000	48	2.29	49.8	1,582	Dog	Oral	—	H
*Porphyromonas levii* ATCC 29147	ARBX00000000	125	2.51	45.7	2,150	Cow	Nonoral—rumen	—	—
*Porphyromonadaceae* COT-184 OH4590	JRAN01000000	79	2.39	37.6	1,859	Dog	Oral	—	—
*Bacteroides fragilis*	NC_016776	1	5.37	43.4	4,290	Human	Nonoral—gut	—	—
*Tannerella forsythia*	NC_016610	1	3.41	47.0	3,001	Human	Oral	—	D
*Paludibacter propionicigenes*	NC_014734	1	3.69	38.9	3,020	Environmental	Nonoral—soil	—	—

### Functional Analysis of Genomes

The genomes for all strains were uploaded to RAST (v2.0) (Aziz et al. 2008; Overbeek et al. 2014) and annotated with the ClassicRAST scheme with automatic error fixing and backfilling of gaps. Annotations were then manually curated to reduce the impact of partial/misassemblies or erroneous gene annotations; this was done using a combination of visual inspection, homology searches, and multiple sequence alignments. The subsequent amino acid sequences and genome annotations were used for downstream analyses.

### Identification of Homologous Clusters

Homologous clusters used in the subsequent analyses of phylogeny and gene content were identified by using a Markov clustering approach. Predicted protein sequences from RAST were first filtered to remove those shorter than 50 amino acids in length. A reciprocal Basic Local Alignment Search Tool (BLAST) was performed, BLASTall (v2.2.25) (-b 10000 -v 10000 -p blastp -e 1e-5) (Altschul et al. 1990). The BLAST results were filtered to remove spurious hits (percentage identity ≥ 25, alignment length ≥ 40, and bit score ≥ 45), then further filtered so that only queries with reciprocal matches were retained. Markov cluster algorithms were applied to group the sequences, MCL (v12.135) ((mcxload –stream-mirror –stream-neg-log10 -stream-tf ‘ceil(200)') and (mcl -I 1.4)) (Li et al. 2003). The resultant clusters were then converted into counts and binary matrices using in house Perl scripts (supplementary tables S1 and S2, Supplementary Material online) and annotated using the consensus annotation within the cluster. Sequences that failed to meet the above criteria or failed to form clusters were excluded from downstream analyses.

### Phylogenetic Analysis

To investigate the phylogenetic relationship between *Porphyromonas* species, core sequence and gene content methods were applied. For the supermatrix method, single copy core sequences were extracted from the set of homologous clusters. The sequences from each cluster were aligned using “muscle” (v3.8.31) (default settings) (Edgar 2004) and then concatenated for each strain. The consequent concatenated alignment was filtered for low-quality sites using Gblocks (v.0.91b) (maximum contiguous nonconserved positions 8, minimum block length 10) (Castresana 2000). The filtered alignments were used to construct a maximum-likelihood (ML) tree using FastTree (v2.1.7) (using a Whelan and Goldman matrix and optimized using Gamma20 likelihood for 1,000 bootstraps) (Price et al. 2010).

### Gene Content Clustering

The binary presence/absence matrix of homologous clusters computed by MCL was used to calculate the Jaccard distance between each strain, using the “dist.binary” function from the R (v3.0.2) package “ade4” (v1.6-2) (Dray and Dufour 2007). The resultant distance matrix was used to create a hierarchical clustering of the strains. The trees from both the supermatrix and gene content methods were plotted using FigTree (v1.4.1) (unpublished) and annotated using Inkscape (v0.48.4) (unpublished). The gene content of strains is also represented as a heatmap calculated from the binary matrix using the binary distance function from R base which was subsequently converted into similarities.

### Average Nucleotide Identity

The average nucleotide identity (ANI) analysis used the assembled whole-genome nucleotide sequence data and followed the method and 95% species criteria as previously reported (Goris et al. 2007; Chan et al. 2012; Kim et al. 2014). For all 32 genomes and for each reciprocal pair, one genome was chosen as a query and the other as a reference. The query was split into consecutive fragments of 500 bp, smaller fragments were discarded. The query fragments were then used to interrogate the reference genome using BLASTn (v2.2.28) (–xdrop_gap_final 150 –penalty -1 –reward 1 –gapopen 3 –gapextend 2 –dust no) (Altschul et al. 1990). For each query if the alignment exhibited at least 70% identity for at least 70% of the query length, the hit with the best bit-score was retained for further evaluation. The ANI score was calculated as the average alignment identity of the retained hits for each reciprocal pair. The ANI scores were used to create a distance matrix to represent the ANI divergence (100% − ANI), this matrix was used to compute a hierarchical clustering using “hclust” function in R (v3.0.2) (method=“complete”) the hierarchical clustering was plotted using “ggplot2” (Wickham 2009) (supplementary fig. S1, Supplementary Material online).

## Results

### Phylogenetic Analysis

**Fig. 1.—:**
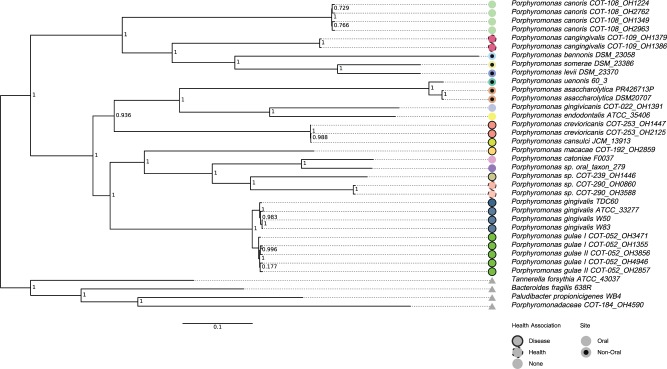
Core sequence ML tree. Supermatrix ML tree inferred from the concatenation of 492 single copy core cluster sequences showing node bootstrap values. Circles represent species groups, colored by species. Triangles are used to represent the four *Porphyromonadaceae* family strains (outgroup). Circles with black insertion were from stains isolated from nonoral sites. Circles with full outlines have known oral disease associations; staggered outlines have associations with oral health; and no outline have no known association.

**Fig. 2.—:**
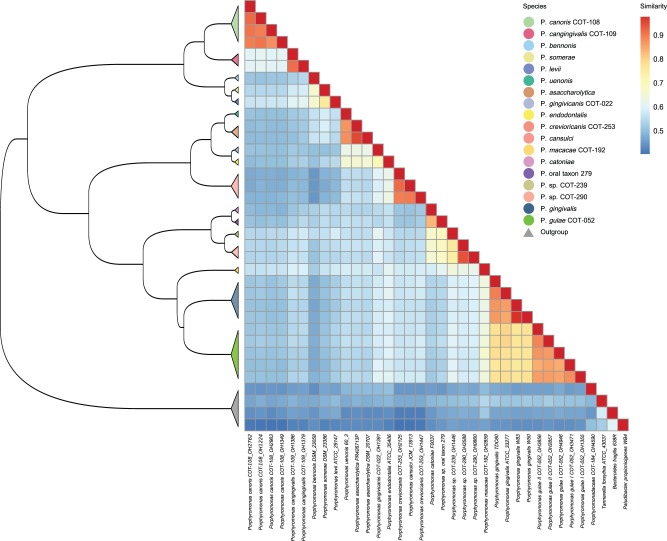
Binary gene content dendrogram and heatmap. The dendrogram was inferred by hierarchical clustering based of dissimilarities of gene content using Jaccard distances. Isolates of the same species are represented by triangles, nodes are colored by species. *Porphyromonas crevioricanis and *Porphyromonas cansulci* have been co-colored as thought to be isoforms of the same species. Heatmap constructed using binary distance (converted to similarity) between strains.

To understand the relatedness of the Porphyromonad strains, two complementary techniques were applied. First, a phylogenetic tree was inferred based on sequences common to all of the strains (core genome). The core genome was constructed for the 32 *Porphyromonas* strains (ingroup) and also for the 36 genome set including the four outgroup species. The ingroup core genome consisted of 657 (18%) core clusters from a pan-genome of 3,751 clusters, 492 of 657 core clusters were identified as single copy. After including the outgroup 560 (13%) of 4,460 were core clusters and of these 363 were single copy. The clusters were aligned separately and then concatenated to form core genomes. The ingroup alignment consisted of 199,261 amino acid positions which reduced to 131,810 (66% average global identity) positions after filtering (supplementary file S1, Supplementary Material online). The outgroup alignment contained 146,429 positions which reduced to 91,552 (66% average global identity) after filtering (supplementary file S2, Supplementary Material online). These alignments were then used to infer a phylogenetic tree (fig. 1). The second method clustered strains based on their shared gene content using the gene cluster matrix. This method did not have the scope to reconstruct evolutionary distance but was able to group strains based on which genes were present or absent using a Jaccard distance matrix (supplementary file S3, Supplementary Material online). The Jaccard distance is the size of the intersection divided by the size of the union subtracted from one; therefore, identical samples would have a pairwise value of zero whereas unrelated samples would have a value of 1. The two most similar pairwise comparisons were between *P. gingivalis* W50 and *P. gingivalis* W83 (0.16) and between *Porphyromonas asaccharolytica* DSM20707 and *P. asaccharolytica* PR426713P (0.23)*.* The two least similar strains were between *Pa. propionicigenes* WB4 and the two *Porphyromonas canoris* strains; OH1554 and OH1349 (0.77 and 0.77). A hierarchical clustering of the pairwise Jaccard distances along with similarity heatmap is presented in figure 2. For both the ML and binary gene content methods, isolates of the same named species were grouped together at the nodes creating monophyletic groups. Canine and human oral strains did not form separate clusters neither did strains classed as health or disease associated. There were divides seen between the ingroup and outgroup strains which separated at the earliest point forming the root, the nonoral strains also separated but formed two disparate groups either side of the root.

### Comparison of Species Relatedness

Given the small distance between *P. gulae* and *P. gingivalis* isolates in the core genome phylogenetic tree (fig. 1), it was of interest to determine whether *P. gulae* and *P. gingivalis* were distinct species or could be reclassified as related subspecies. ANI values were calculated, pairwise ANI comparisons between *P. gingivalis* isolates gave values between 98.5% and 100%. Between *P. gulae* isolates the pairwise ANI values were between 98.0% and 98.3% and when *P. gingivalis* isolates were compared with *P. gulae* isolates the pairwise ANI values were between 92.7% and 92.9% (supplementary table S3, Supplementary Material online).

*Porphyromonas crevioricanis* and *Porphyromonas cansulci* were also closely related (fig. 1) and have previously been reported to be very similar if not the same species (Sakamoto and Ohkuma 2013; Sakamoto et al. 2013). Pairwise ANI calculations between these strains were performed and the ANI between the two *P. crevioricanis* strains and one *P. cansulci* strain was 99.5% (supplementary table S3, Supplementary Material online).

### Metabolic Processes that Differ between *P. cangingivalis* and Other Porphyromonads

#### Iron

The role of iron and its regulation and acquisition in *P. gingivalis* has been studied extensively. Based on previous research 21 iron-related genes were identified in the *P. gingivalis* strains and then their presence or absence in the 16 other Porphyromonad species was determined (table 2). The genes fell into two main groups, the first group comprised genes conserved in all or almost all of the species examined. These included a *tonB* homolog putatively involved in ferric siderophore transport, ferritin-like protein (iron storage), ferrochetalase, and a ferrous iron transport protein (table 2). The other main group was present largely in only *P. gingivalis* and *P. gulae.* These included various hemagglutins, two hemolysins, two arginine-specific proteases (*RGPA* and *RGPB*) and a hemophore-like protein *HusA*. Outside of these two groups, a ferric uptake regulator (*Fur*) was found in *P gingivalis*, *P. gulae* and some of the oral human isolates but in none of the canine isolates. In addition, two iron compound ATP-binding cassette (ABC) transporter proteins were only found in *P. gingivalis*, *P. gulae,* and *P. cangingivalis*. Finally, lysine protease KGP was only found in *P. gingivalis* as were most of the hemagglutinins.

**Table 2:** Genes Involved in Iron Acquisition, Regulation, or Utilization

	HagA ref: YP_004508794	HagB ref: YP_004508981	HagC ref: YP_004508984	HagD gbk: AAB49691	HagE gbk: AAQ66991	Hemagglutinin gbk: WP_012458040	Hemagglutinin gbk: WP_005873402	Hemolysin ref: WP_023847110	Hemolysin ref: WP_005873396	Cell Envelope Biogenesis Protein TonB ref: WP_013815139	TonB-Dependent Receptor ref: WP_013816293	Ferritin ref: WP_004585574	Ferrochelatase ref:WP_013815385	Iron Transport FeoB ref:WP_013816689	Iron ABC Transporter ref:WP_013816382	Iron ABC Transporter Substrate-Binding Protein ref:WP_013816383	Fur Family Transcriptional Regulator ref:WP_004584994	Arginine-Specific Protease RGPA	Arginine-Specific Protease RGPB	Lysine-Specific Protease KGP	HusA ref:WP_005874769
	Hemagglutanins							Hemolysins						Iron Transport				Gingipains			
*Porphyromonas canoris*										+		+	+	+							
*Porphyromonas cangingivalis*										+	+	+	+	+	+	+					
*Porphyromonas bennonis*										+			+	+							
*Porphyromonas somerae*										+		+	+	+							
*Porphyromonas levii*										+		+	+	+							
*Porphyromonas uenonis*										+	+	+	+	+			+				
*Porphyromonas asaccharolytica*										+	+	+	+	+			+				
*Porphyromonas gingivicanis*						+				+	+	+	+	+							
*Porphyromonas endodontalis*										+	+	+	+	+			+				
*Porphyromonas crevioricanis*								+		+	+	+	+	+							
*Porphyromonas catoniae*										+	+	+	+	+	+						
*Porphyromonas* sp. oral taxon 279										+	+	+	+	+							
*Porphyromonas* sp. COT-239										+	+	+	+	+	+						
*Porphyromonas* sp. COT-290										+	+	+	+	+							
*Porphyromonas macacae*										+	+	+	+	+							
*Porphyromonas gingivalis*	+	+	+	+	+	+	+	+	+	+	+	+	+	+	+	+	+	+	+	+	+
*Porphyromonas gulae*		+				+	+	+		+	+	+	+	+	+	+	+	+	+		+

#### Protoporphyrin IX

To identify genes that might confer metabolic flexibility, metabolic pathways that contained genes which differed between *P. cangingivalis* and the other Porphyromonads were analyzed. Given the importance of heme and its likely limited availability in healthy gingiva, the genes required for protoporphyrin IX synthesis were promising candidates and therefore investigated along with other related pathways.

**Fig. 3.—:**
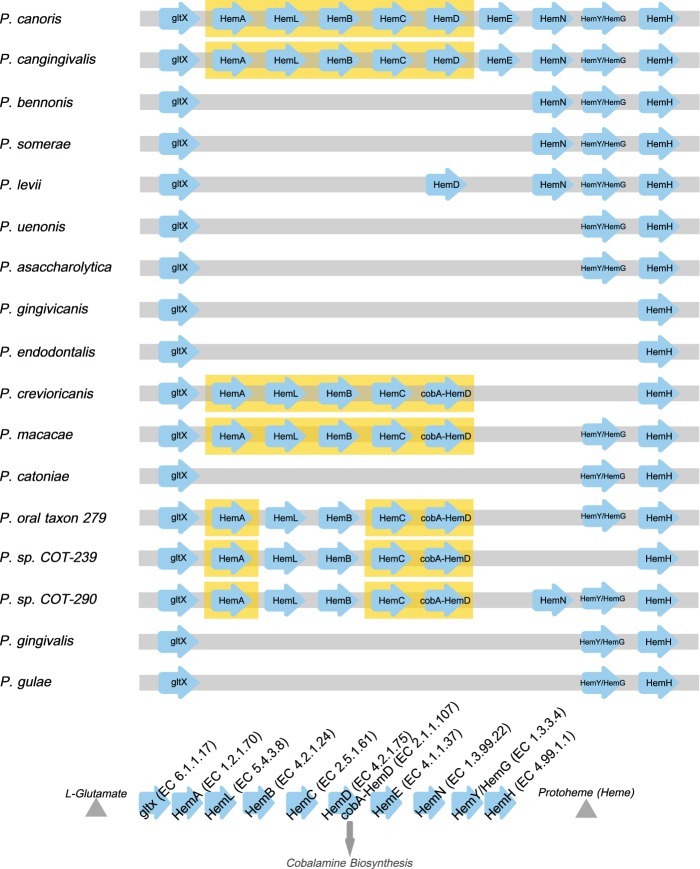
The protoporphyrin IX synthesis pathway. Arrow icons indicate individual enzymes ordered by the pathway order, missing icons indicate missing enzymes from the genomes and yellow boxing highlights enzymes forming clusters. Schematic represents pathway order.

The metabolic pathway for protoporphyrin IX synthesis consists of ten enzymes that catalyze the conversion of L-glutamate to protoheme (heme) (fig. 3). Figure 3 is a schematic of the presence of these enzymes within the genomes of the *Porphyromonas* species. The first (gltx) and last (HemH) enzymes of the pathway were annotated in all 17 species, the penultimate enzyme of the pathway HemY/HemG was present in 13 species whereas the remaining seven enzymes were absent from the majority of the species. The only species to have all ten enzymes annotated within their genomes were *P. cangingivalis* and *P. canoris*. There were five species that contained a partially complete pathway these were P*.* sp. COT-290, *P.* sp. COT-239, *P.* oral taxon 279, *Porphyromonas macacae,* and *P. crevioricanis*, this group contained all of the enzymes required to convert L-glutamate to uroporphyrinogen III.

**Fig. 4.—:**
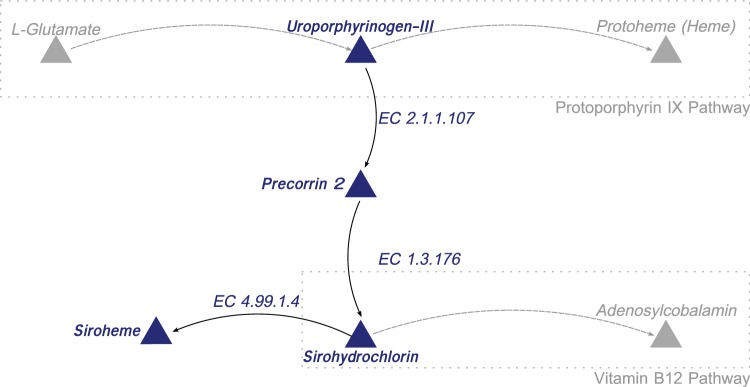
Links between the protoporphyrin IX, siroheme and vitamin B12 synthesis pathways. The figure shows the connection between the protoporphyrin IX pathway and vitamin B12 synthesis pathway. Staggered lines represent a summary of the pathway, full lines represent specific reactions, arrows indicate pathway direction, and compounds are represented by triangles.

**Fig. 5.—:**
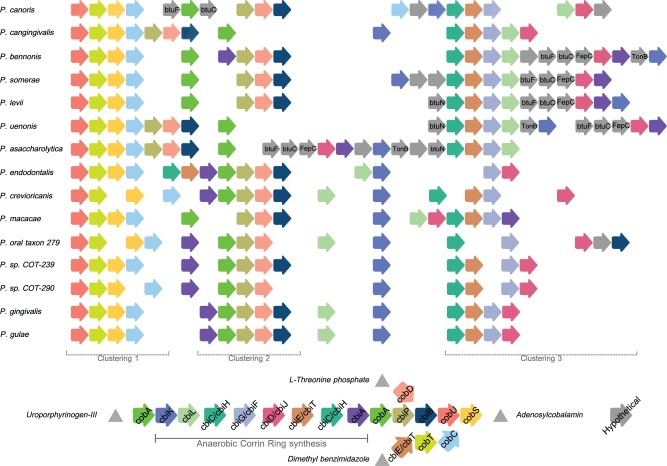
The anaerobic vitamin B12 synthesis pathway. Colored arrow icons indicate individual enzymes grouped by clustering, hypothetical or nonpathway enzymes are gray and named where possible. Missing icons indicate enzymes not found within the genomes. Schematic represents pathway order.

The pathway for protoporphyrin IX branches at uroporphyrinogen III leading to the synthesis of two other critical enzymatic cofactors siroheme and vitamin B12 (fig. 4). Investigating these pathways revealed further differences between the Porphyromonad species. Out of all of the 32 genomes examined, only *P. cangingivalis* possessed siroheme synthase and was therefore the only Porphyromonad that had the capacity to synthesize siroheme from uroporphyrinogen III (fig. 4). The pathway for synthesizing vitamin B12 (cobalamin) is complex and branches from the siroheme pathway with which it shares *cobA* (figs. 4 and 5). Of the 17 Porphyromonads analyzed, the genomes of 13 species contained all of the genes required to synthesize cobalamin from uroporphyrinogen III regardless of whether they had the genes to synthesize uroporphyrinogen III or not. The only exceptions to this were *P. cangingivalis* and *P. canoris,* which were missing *cbiA* (cobyrinic acid a,c-diamide) (fig. 5) and *Porphyromonas gingivicanis* and *Porphyromonas catoniae*, which lacked the entire pathway.

As the starting molecule for protoporphyrin IX synthesis is glutamate, it was feasible that *P. cangingivalis* might have an increased glutamate requirement and therefore the genes relating to glutamate synthesis were examined. Analysis of genes relating to glutamate synthesis identified several differences between *P. cangingivalis* strains and other Porphyromonads. *Porphyromonas cangingivalis* strains contained three genes involved in converting nitrite to ammonia (a precursor of glutamate) that were absent in *P. gulae* (table 3). In addition the genome of *P. cangingivalis* contained an NADP(H)-dependent glutamate dehydrogenase that could synthesize glutamate from ammonia. In contrast, *P. gulae* had an NAD(H)-dependent glutamate dehydrogenase that could catabolize glutamate to ammonia (table 3). *Porphyromonas cangingivalis* also lacked aspartate amino transferase (AAT) which either synthesizes glutamate from aspartate and α-ketoglutarate or in the reverse direction generates aspartate from glutamate and oxaloacetate. Finally, three genes were identified that had the potential to supply the extra NADPH required by glutamate dehydrogenase in *P. cangingivalis.* These genes make up the oxidative phase of the pentose phosphate pathway that generates two NADPH molecules and were present in *P. cangingivalis* strains but not in *P. gulae* strains (table 4).

**Table 3:** Glutamate Enzymes

Species	Cytochrome *c* Biogenesis Protein CcsA	Cytochrome *c* Nitrite Reductase, Small Subunit NrfH	Cytochrome c552 Precursor NrfA (EC 1.7.2.2)	Aspartate Aminotransferase (EC 2.6.1.1)	NAD-Specific Glutamate Dehydrogenase	NADP-Specific Glutamate Dehydrogenase
					(EC 1.4.1.2)	(EC 1.4.1.4)
*Porphyromonas canoris*	+	+	+	+		+
*Porphyromonas cangingivalis*	+	+	+			+
*Porphyromonas bennonis*	+	+	+			
*Porphyromonas somerae*						+
*Porphyromonas levii*						+
*Porphyromonas uenonis*						+
*Porphyromonas asaccharolytica*						+
*Porphyromonas gingivicanis*					+	
*Porphyromonas endodontalis*					+	
*Porphyromonas crevioricanis*				+	+	
*Porphyromonas catoniae*						+
*Porphyromonas* sp. oral taxon 279	+		+			+
*Porphyromonas* sp. COT-239						+
*Porphyromonas* sp. COT-290	+	+	+			+
*Porphyromonas macacae*	+	+	+	+	+	
*Porphyromonas gingivalis*	+	+	+	+	+	
*Porphyromonas gulae*				+	+

**Table 4:** Pentose Phosphate Enzymes

	Glucose-6-Phosphate1-Dehydrogenase(EC 1.1.1.49)	6-Phosphogluconate Dehydrogenase, Decarboxylating(EC 1.1.1.44)	6-Phosphogluco-Nolactonase(EC 3.1.1.31)
	G6PD	PGD	PGLS
*Porphyromonas canoris*	+	+	+
*Porphyromonas cangingivalis*	+	+	+
*Porphyromonas bennonis*			+
*Porphyromonas somerae*			+
*Porphyromonas levii*			
*Porphyromonas uenonis*			+
*Porphyromonas asaccharolytica*			+
*Porphyromonas gingivicanis*			+
*Porphyromonas endodontalis*			+
*Porphyromonas crevioricanis*			
*Porphyromonas catoniae*	+	+	+
*Porphyromonas* sp. oral taxon 279	+	+	+
*Porphyromonas* sp. COT-239	+	+	+
*Porphyromonas* sp. COT-290	+	+	+
*Porphyromonas macacae*	+	+	+
*Porphyromonas gingivalis*			
*Porphyromonas gulae*			

## Discussion

### Phylogenetic Analysis

The core genome by definition is a pool of genes common to all members of a group and considered integral to fundamental aspects of biology and phenotypic traits (Medini et al. 2005). The genes within the core are therefore involved in functions, such as regulation, metabolism, and cell division. This is seen in the core set identified within the *Porphyromonas* genomes, which includes genes such as *MutL*, *RpoN,* and *RnfB*. It has been argued that core genes are recalcitrant to transfer (Eisen 2000) and stable over time as recipient taxa would likely already contain orthologs of similar function. However, there is some evidence to suggest that certain genes defined as being of core function are liable to core gene transfer (Everitt et al. 2014), which would have the potential to obscure the results of a core gene phylogeny.

The ML tree inferred using the core gene set roots at the midpoint, separating *P. canoris, P. cangingivalis, P. bennonis, Porphyromonas somerae,* and *Porphyromonas levii* from the other *Porphyromonas* species (fig. 1). This separation has also been demonstrated previously using analysis of 16S rDNA gene sequences (Fournier et al. 2001; Summanen et al. 2009, 2005; Gronow et al. 2011). The nonoral isolates (isolated from a skin abscess, leg ulcer, and bovine rumen) have been described as opportunistic pathogens, with their presence reported in clinical infections in a number of studies (Finegold et al. 2004; Summanen et al. 2005; Sweeney et al. 2009). In contrast, *P. cangingivalis* and *P. canoris* are among the most abundant *Porphyromonas* species in the canine oral environment (quantified by 16S rDNA sequences) (Davis et al. 2013), with *P. cangingivalis* being the most abundant of all canine oral species, with a high prevalence in plaque from healthy gingiva as well as plaque during periodontitis (Davis et al. 2013)*.* This topology is repeated in the gene content clustering (fig. 2), suggesting that these species not only had an early split from the remaining species inferred from the ML tree but have core differences in gene content and therefore protein-coding capacity.

The remaining nonoral strains *P. asaccharolytica* and *Porphyromonas uenonis* form a clade, confirming previous observations using 16S rDNA genes (Finegold et al. 2004; Mikkelsen et al. 2008). *Porphyromonas asaccharolytica* particularly has been associated with pathogenesis at sites in addition to the oral cavity (Sharma and Rouphael 2009; Caiano Gil et al. 2013; Takeda et al. 2013). *Porphyromonas asaccharolytica* and *P. uenonis* have been shown to be biochemically similar (Finegold et al. 2004) to the oral associate *Porphyromonas endodontalis* (Cao et al. 2012; Lombardo Bedran et al. 2012), which is a sister clade to *P. asaccharolytica* and *P. uenonis*. Bifolious to *P. endodontalis* is *P. gingivicanis* a novel canine strain (Hirasawa and Takada 1994). Next to this clade are *P. crevioricanis* and *P. cansulci* which were confirmed by pairwise ANI comparisons (99.5%) to be the same species (supplementary table S3, Supplementary Material online). Strains of the human periodontitis-related species *P. gingivalis* (Pihlstrom et al. 2005) form a monophyletic group that is similar but distinct from the putative canine periodontitis *P. gulae* strains (Nomura et al. 2012; Yamasaki et al. 2012).

The ML tree is mainly in agreement with previously published studies looking at 16S rDNA genes in isolation, and this topology is recapitulated in the gene content of the strains as seen in the gene content clustering. The tree topology however is not entirely concordant with some phenotypic differences observed within the genus. For example, it has been previously reported from biochemical tests (Fournier et al. 2001; Mikkelsen et al. 2008) that approximately half of the Porphyromonad species test positive for catalase (Mikkelsen et al. 2008), an enzyme commonly found in aerobic bacteria that detoxifies molecular oxygen. This observation was confirmed here in silico by the identification of the catalase (EC 1.11.1.6) enzyme. However, the distribution of species containing catalase does not fall where one would expect for a single point of origin. For example, it is absent from *P. gingivalis*, *P. somerae* and *P. levii* but present in *P. gulae, P. cangingivalis* and *P. canoris.* This would suggest that through parsimony catalase has been acquired or lost in multiple events; the acquisition of catalase was supported by looking at the neighboring genes which were different for each species. A phylogeny inferred using the catalase genes found within the canine species did not resemble the phylogeny shown by the core gene set (supplementary fig. S2, Supplementary Material online). Furthermore, catalase is absent from all of the human oral isolates but present in all (with the exception of *P. crevioricanis*) of the dog species. This would advocate that catalase provides an advantage to bacteria living within canine mouths. It is therefore plausible that canine Porphyromonads have acquired catalase from other bacteria within the mouth, this is consistent with previous reports where similar results were attributed to multiple migrations of catalase (Fast et al. 2003; Passardi et al. 2007). The value of catalase may be enhanced by its high turnover rate (Chidrawi et al. 2008), with its high level of enzyme activity magnifying the advantage of incorporation. The presence of catalase may help canine Porphyromonads to tolerate higher levels of oxygen in the environment potentially allowing them to become more prevalent in healthy plaque than are their human cousins.

The close relationship between *P. gingivalis* and *P. gulae* isolates was investigated further to determine whether the two are from different species. *Porphyromonas gulae* was first described as a separate species largely as a result of DNA–DNA hybridization results (Fournier et al. 2001). However, the 16S rDNA sequence data in the same study were less clear cut. In this study, ANI values (Goris et al. 2007) were calculated. When the *P. gulae* and *P. gingivalis* isolates were compared (supplementary table S3, Supplementary Material online) the pairwise ANI values were in the range 92.7–92.9%. Based on a previously determined threshold of 95% or above for isolates being from the same species (Chan et al. 2012; Kim et al. 2014) this confirms that despite their high level of similarity *P. gulae* and *P. gingivalis* are sufficiently different to be considered separate species.

### Adaptations of *P. cangingivalis* for Proliferating in Health

*Porphyromonas cangingivalis* is the most prevalent canine oral bacterium found in 16S rDNA studies. It therefore has properties that make it able to persist in the canine oral cavity, particularly in healthy plaque where it averages over 10% of the bacteria present (Davis et al. 2013). Differences between *P. cangingivalis* and other Porphyromonads were identified that are consistent with what we already know about the likely conditions present in healthy plaque compared to during periodontal disease; a number of linked metabolic pathways were analyzed that all center on the production of heme.

### Heme

One of the key differences for bacteria seeking to proliferate in plaque from healthy gingiva compared with the plaque associated with periodontal disease is the availability of various nutrients such as sources of energy and cofactors like heme. In Porphyromonads, heme is thought to be acquired predominantly from the breakdown of hemoglobin from blood (Amano 1995; Nhien et al. 2010). Many other bacteria do however contain the machinery to synthesize their own heme (Hansson et al. 1991; McNicholas et al. 1997; Hashimoto et al. 1997; Guégan et al. 2003; Xiong et al. 2007), which offers an alternative strategy to heme acquisition particularly when this is limited in an environment. The relationship between sourcing heme endogenously or exogenously is not fully understood, but may center on the energy expense of synthesizing rather than foraging (Anzaldi and Skaar 2010). Heme is not only important for growth but also has important roles in defense and virulence (Olczak et al. 2005), Porphyromonads in general are black pigmented because they store heme at the cell surfaces in vast quantities. This is thought to provide some level of protection from peroxidases generated as part of the host’s immune response (Smalley et al. 2000).

*Porphyromonas gingivalis* has an array of genes which are important for acquiring and utilizing iron (Olczak et al. 2008). The presence or absence of many of these heme- and iron-related proteins was analyzed across the various Porphyromonad genomes studied here. First a key difference between *P. gulae* (and other Porphyromonads) and its human counterpart *P. gingivalis* was observed, despite most of the iron-related genes being the same between the two strains, a copy of the lysine gingipains gene (KGP) was not identified in any *P. gulae* strain. In contrast, both of the arginine gingipains genes (*RGPA* and *RGPB*) were present. The gingipains genes have been heavily studied and shown to be important for *P. gingivalis* virulence. The genes encode for outer membrane cysteine proteases that are required for host colonization, inactivation of host defenses, tissue destruction, and modulation of the host immune system (Holt et al. 1999). Gene knockout experiments have shown that a lysine gingipains mutant of *P. gingivalis* had reduced heme agglutination and increased sensitivity to complement compared with its wild-type parent (Grenier et al. 2003), which results from an increased sensitivity to damage by H_2_O_2_. Given that none of the canine Porphyromonads have a lysine gingipains gene and only *P. gulae* has arginine gingipains genes, it might be supposed that the canine Porphyromonads have an increased sensitivity to H_2_O_2_. However as already mentioned, all the canine Porphyromonads (except *P. crevioricanis*) have a catalase gene that may protect against H_2_O_2_. Therefore it is feasible that *P. gulae* has lost the lysine gingipains gene because it has become redundant, with its main function (protection against H_2_O_2_) now being provided by catalase.

Comparing the iron-related genes from *P. cangingivalis* with those from other Porphyromonads showed that many are retained in *P. cangingivalis*. Genes for iron transport and iron storage (ferritin) are all present (table 2). In contrast to this, the genes for hemagglutination, the arginine gingipains genes, and the ferric uptake regulator gene are missing. *Porphyromonas cangingivalis* may therefore still have the ability to uptake heme, potentially as a source of iron, but the absence of hemagglutinating genes in *P. cangingivalis* compared with *P. gulae* may relate to a divergence in lifestyle. Assuming the latter retains the pathogenic properties of *P. gingivalis*, an important part of its mechanism of action may be to attach to and invade canine gingival tissue cells. If *P. cangingivalis* has evolved to a commensal modality, there may be a disadvantage to attacking canine cells that might elicit an inflammatory response. Under those circumstances the loss of the ability to hemagglutinate red blood cells may be a strategy to prevent attachment to canine gingival cells and the concomitant initiation of inflammation. This may be particularly important in a healthy plaque environment where stimulating the inflammatory response will lead to the generation of altered physiological conditions that other species (particularly other Porphyromonads) may be better adapted to utilize. Other gene differences between *P. cangingivalis* and *P. gulae* also appear to relate to their likely exposure to iron. *Porphyromonas cangingivalis* lacks *HusA* which is a hemophore-like protein that has been previously described to have a role in the uptake of heme from gingipains in *P. gingivalis* (Gao et al. 2010). *HusA* is expressed when heme levels are very low as it has a high affinity for heme and can effectively scavenge it from the environment. In contrast, *P. cangingivalis* may not need to scavenge heme under limiting conditions as it may be able to synthesize its own from protoporphyrin IX and iron.

The ferric uptake regulator homolog (*Har*) in *P. gingivalis* (Butler et al. 2014) regulates a number of genes in response to heme and has a role in biofilm formation in a heme responsive manner. Within the canine *Porphyromonads Har* is only present in *P. gulae* (table 4). Similarly the loss of ferric uptake regulator (*Fur*) has been reported in the genus *Rickettsia* and attributed to obligatory intracellular milieu (Panek and O’Brian 2002). It may therefore be possible that health associated Porphyomonad species have dispensed with gene regulation of heme uptake because they are likely to be operating in conditions where heme levels are low or alternatively are using a currently unidentified novel ortholog.

In a healthy mouth where blood is not present, iron will be complexed to the salivary proteins lactoferrin and transferrin. These are host scavenging systems that seek to reduce the iron available to oral bacteria as an antimicrobial defense. Some bacteria have been shown to subvert the host defense by being able to import transferrin and its associated iron (Banerjee et al. 2012). Examples of bacteria that are able to make use of iron bound within transferrin and lactoferrin include Neisseriaceae and Pasteurellaceae (Ratledge and Dover 2000), within these families of bacteria are other canine oral health-associated species such as *Neisseria shayeganii* and *Pasteurella dagmatis* (Davis et al. 2013; Holcombe et al. 2014). Studies have shown that the genes required to uptake host expressed siderophores are generally expressed in operons of genes which include; a linked outer membrane receptor, a periplasmic-binding protein (PBP), and an inner membrane ABC transporter (Krewulak and Vogel 2008). Within the iron-related genes analyzed in table 2, a PBP protein and an ABC transporter were found to be both present in only *P. cangingivalis, P. gulae,* and *P. gingivalis*. This may be part of a siderophore scavenging system used by *P. cangingivalis* in healthy plaque, although that does not explain the presence of the same genes found in *P. gingivalis* and *P. gulae*. It is however feasible that genes related to iron uptake that are unique to *P. cangingivalis* may remain undetected through lack of annotation. Assuming that *P. cangingivalis* is able to acquire iron, it also requires a source of protoporphyrin IX to be able to synthesize heme. A complete protoporphyrin IX synthesis pathway is present in *P. cangingivalis* and the same pathway is also present to varying degrees across the other Porphyromonads (fig. 3), which is suggestive that those species without a complete pathway are able to synthesize protoporphyrin IX by foraging precursors liberated by other species with more intact pathways.

The presence of the heme synthesis pathways is well documented across the tree of life, likewise is its loss (Panek and O’Brian 2002; Kořený et al. 2013). The loss of the pathway may arise due to degeneration of genes that are no longer under selective pressure, for example, when heme is readily available within a host environment. The loss of the pathway may also be more directly advantageous to bacteria by reducing the potential of cell damage from reactive oxygen species produced by the accumulation of heme (Anzaldi and Skaar 2010). Along with examples of the heme pathway being lost there are also examples of the pathway being regained as new niches are explored, for example, the group Kinetoplastea (Korený et al. 2010; Alves et al. 2011) which have regained partial or full pathways despite a complete absence within their ancestors. Whether the Porphyromonads have lost, gained, or lost and regained the heme pathway is unclear, but the completeness of the heme pathway may be reflective of the evolutionary strategy employed by the bacteria. Bacteria such as *P. cangingivalis* have maintained a complete (or near complete) pathway and therefore remained independent of the host, whereas more pathogenic bacteria such as *P. gingivalis* may have discarded their machinery in favor of exogenous heme acquisition, which requires less energy and therefore enables rapid growth when environmental conditions are favorable.

### Other Pathways Linked to Protoporphyrin IX

Branching off the protoporphyrin IX pathway are genes for the synthesis of two other key cofactors, siroheme and vitamin B12 (cobalamin). Of all the Porphyromonads studied, only *P. cangingivalis* has the siroheme synthase gene. Siroheme has been found to be associated with two different classes of enzymes—nitrite and sulfite reductases (Krewulak and Vogel 2008). This raises the possibility that the nitrite reductase identified as potentially helping to supply glutamate in *P. cangingivalis* requires siroheme.

Vitamin B12 is required by all bacteria and the majority of the Porphyromonads has the second half of the pathway from precorrin 2 onwards (fig. 4). The exceptions are *P. gingivicanis* and *P. catoniae* that lack the entire pathway (and presumably scavenge vitamin B12) and *P. cangingivalis* and *P. canoris* that appear to lack CbiA. Given that these species have all the other genes (19 in total) required for synthesizing vitamin B12 from glutamate, it is possible that CbiA function in these strains is performed by another gene. Of particular interest is that *P. cangingivalis* uniquely among porphyromonads appears to have the genes required to make the CobN cobalt chelatase. This enzyme is normally part of the aerobic route to vitamin B12 synthesis and encodes the step in the pathway immediately before the anaerobic and aerobic pathways converge (Rodionov et al. 2003). One possibility is that *P. cangingivalis* has acquired this gene complex to allow it to scavenge intermediates from its aerobic neighbors which could be an advantage in the more oxygen rich environment of healthy gingiva.

### Generating a Supply of Glutamate

To ensure an adequate amount of protoporphyrin IX can be synthesized, the starting precursor molecule(s) need to be present in sufficient amounts. The protoporphyrin IX pathway requires glutamate as a precursor (fig. 3). *Porphyromonas cangingivalis* therefore possesses a number of related genes that supply the building blocks and the energy required to provide a ready supply of glutamate. Three genes were identified which are homologs of three components of a cytochrome C nitrite reductase (*NrfA*, *NrfH*, and *Cytochrome C biogenesis protein CcsA*; Kranz et al. 2009; Einsle 2011). The combined action of these enzymes converts nitrite to ammonia by combining it with formate. Nitrite is readily available in saliva through the action of various bacteria converting nitrate into nitrite (Duncan et al. 1995). Reservoirs of nitrate reducing bacteria have been found on the human tongue (Duncan et al. 1995). To make use of the ammonia generated by nitrite reductase, it must be converted to glutamate. In *P. cangingivalis,* this may be achieved by the action of NADPH-dependent glutamate dehydrogenase. A related enzyme is also present in *P. gulae*. However, the *P. gulae* gene is an NADH-dependant glutamine dehydrogenase that catalyzes the opposite reaction, that is, the degradation of glutamate to ammonia. This gene correlates well with canine oral pathogenesis, being present in all of the disease-associated Porphyromonads (except COT-239) and none of the health-associated ones (table 3). This potentially reflects the differing requirements in the two health states, with amino acids freely available during periodontitis (from proteolytic degradation of host proteins and tissues) but in shorter supply in healthy gingiva with an increased requirement for glutamate to feed protoporphyrin IX synthesis.

The use of an NADPH requiring glutamine dehydrogenase places an increased demand on NADPH levels as an energy source. Perhaps to solve this problem *P. cangingivalis* has three extra genes in the pentose phosphate pathway (table 4). These genes form the oxidative phase of the pathway that generates two extra NADPH molecules for every glucose 6-phosphate molecule processed. In addition to further bolster the glutamate pool *P. cangingivalis* has lost the gene for AAT, which converts aspartate to glutamate and back. In an environment where glutamate levels are high, AAT will favor the glutamate catabolic route, so deletion of this gene possibly allows the generation of a higher concentration of glutamate. Of course, protoporphyrin IX is not the only destination for glutamate in the bacterial cell. Glutamate is a precursor for the synthesis of several amino acids. Therefore, the genes listed above that drive increased glutamate levels may also have a role in supporting the synthesis of amino acids which again is consistent with an environment where sources of amino acids are in short supply.

## Conclusions and Summary

In an attempt to understand the metabolic flexibility of *P. cangingivalis* that might explain its prevalence in the canine mouth both in plaque from healthy gingiva and plaque associated with the development of periodontal disease, genes relating to the generation or acquisition of heme have been analyzed. Even within this limited field of study many genes have been identified which could give *P. cangingivalis* an advantage within the canine oral environment. These differences are summarized in table 5. Along with *P. canoris*, *P. cangingivalis* is the only Porphyromonad to have all the genes required to synthesize protoporphyrin IX, which is potentially required in the absence of blood as a source of heme. Linked to protoporphyrin IX a number of genes were identified that point to the need to generate elevated levels of glutamate which could be used as a precursor for protoporphyrin IX. *Porphyromonas cangingivalis* is the only species with the gene to synthesize siroheme and the only species potentially able to scavenge from the aerobic pathway for vitamin B12 synthesis in other bacteria.

**Table 5:** Summary of Adaptations of *Porphyromonas cangingivalis* for Proliferating in Health

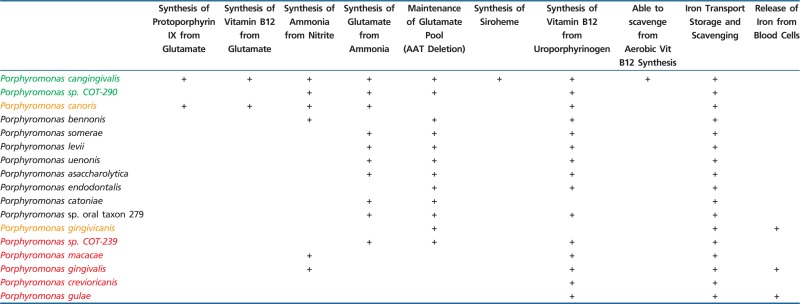

Note.—Red: periodontitis associated in dog; yellow: no association in dog; green: health associated in dog; black: noncanine species. Associations are taken from Davis et al. (2013).

Perhaps just as important are the genes that *P. cangingivalis* lacks. The gingipains genes and most of the hemagglutinin genes are unique to *P. gulae* and *P. gingivalis* and are not found in any other Porphyromonads. These have been shown in *P. gingivalis* to be key virulence factors linked to survival, degradation of host tissues and invasion of host cells. This suggests that the other Porphyromonads may not have a similar role and whether they are disease associated, such as *P. macacae* and *P. crevioricanis*, or just prevalent in disease such as *P. cangingivalis*, they may be responding to pathogenic events rather than initiating them. It might be considered somewhat ironic that *P. cangingivalis*, which has dispensed with many of the reported Porphyromonad virulence genes (e.g., hemolysins, hemagglutinins and gingipains) allowing a more commensal lifestyle, is actually as prevalent in early periodontitis as its cousin *P. gulae* which has these virulence genes. This may relate to the sequential nature of disease development with disease biofilms developing from healthy ones. We therefore speculate that *P. cangingivalis* dominates in healthy biofilms thanks to various metabolic adaptations that allow it to prosper such as the ability to synthesize its own heme. Establishing a strong foothold in a biofilm may be a good way to ensure continued success even when the environmental conditions change as periodontitis develops.

## Supplementary Material

Supplementary files S1–S3, figures S1 and S2, and tables S1–S3 are available at *Genome Biology and Evolution* online (http://www.gbe.oxfordjournals.org/).

Supplementary Data
