# Supplementary material for: Comparative Genomics of the Genus Porphyromonas Identifies Adaptations for Heme Synthesis within the Prevalent Canine Oral Species Porphyromonas cangingivalis
Source: Genome Biol Evol. 2015 Nov 13;7(12):3397–413. doi: 10.1093/gbe/evv220 (PMC4700951; doi:10.1093/gbe/evv220)
Supplement: Supplementary Data [file supp_7_12_3397__index.html]

Comparative Genomics of the Genus Porphyromonas Identifies Adaptations for Heme Synthesis within the Prevalent Canine Oral Species Porphyromonas cangingivalis — Comparative Genomics of the Genus Porphyromonas Identifies Adaptations for Heme Synthesis within the Prevalent Canine Oral Species Porphyromonas cangingivalis — Supplementary Data 

# Comparative Genomics of the Genus *Porphyromonas* Identifies Adaptations for Heme Synthesis within the Prevalent Canine Oral Species *Porphyromonas cangingivalis*

## Supplementary Data

files

- Supplementary Data - zip file
