# Supplementary figures and images for: Comparative Genomics of the Genus Porphyromonas Identifies Adaptations for Heme Synthesis within the Prevalent Canine Oral Species Porphyromonas cangingivalis
Source: Genome Biol Evol. 2015 Nov 13;7(12):3397–413. doi: 10.1093/gbe/evv220 (PMC4700951; doi:10.1093/gbe/evv220)

# Porphyromonas ANI Dendrogram

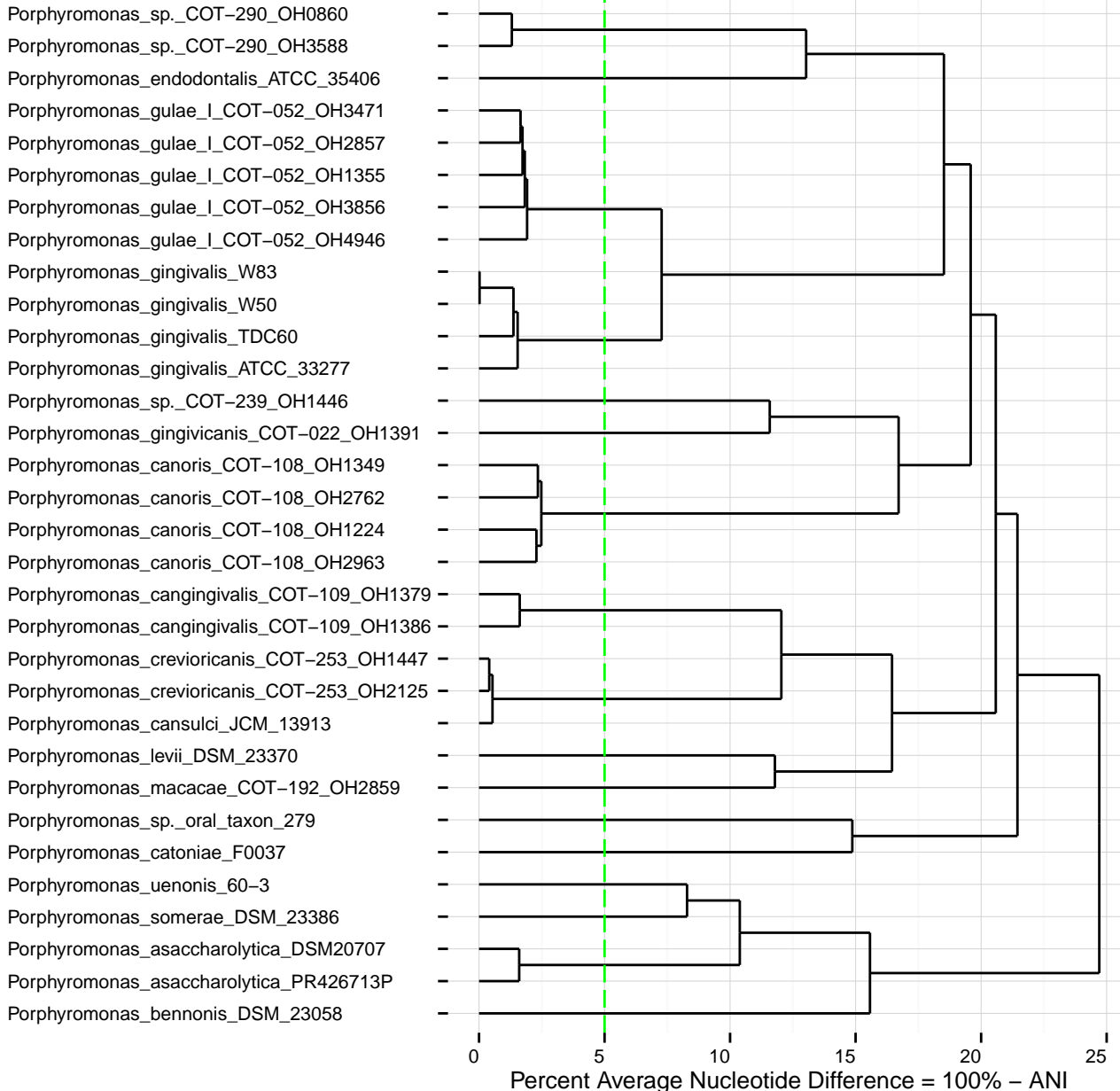

Supplement: Supplementary Data [file supp_evv220_suppl_data.zip › Sup.Figure1.pdf]

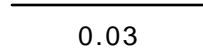

Supplement: Supplementary Data [file supp_evv220_suppl_data.zip › Sup.Figure2.pdf]
